# The Treatment of the Intestinal Disorders of Children

**Published:** 1873-09

**Authors:** S. Henry Dessan

**Affiliations:** Corresponding Member; Attending Physician to the New York Dispensary for the Department of Children’s Diseases; Member of the New York County Medical Society, etc.


### THE TREATMENT OF THE INTESTINAL DISORDERS OF CHILDREN.

CONTRIBUTED TO THE MACON MEDICAL ASSOCIATION,

BY S. HENRY DESSAN, M.D.,

Corresponding Member; Attending Physician to the New York Dispensary for the Department of Children's Diseases; Member of the New York County Medical Society, etc.

*Mr. President, and Gentlemen of the Macon Medical Association:*

Since my last communication to your honorable body, much time has elapsed, owing to change of residence on my part, together with the especial desire of extending my observations and experience in a new field. Having now been connected for about two years with the New York Dispensary, the oldest and largest institution of the kind in this metropolis, and having served in the department of that institution for the Diseases of Children, during my entire connection therewith, I trust that you will at once recognize the motive that induced me to withhold further communications from your learned Society, until I had been able to gather material of sufficient importance to lay before you. I refer more particularly to my connection with the New York Dispensary, as a young physician has very few opportunities elsewhere, during the first decade of his professional life in a large city, for experience and scientific observation.

With these few preparatory remarks, I beg leave to introduce to your attention, "The Treatment of the Intestinal Disorders of Children," as practiced by your contributor in the New York Dispensary. Although indigestion cannot be called an *intestinal* disorder, yet there is so much influence, depending upon disordered function of the stomach in children, over intestinal diseases, that I may be pardoned in saying a few words first concerning this subject.

Of the acute indigestions of nursing children, very few cases, comparatively, are seen in dispensary practice. When they do fall under observation, however, the cause is always inquired into, and, if possible, corrected. If, for instance, overnursing, to prevent crying, be the source of the trouble, more systematic nursing is advised; say give the breast every three hours, and if the child cry in the meantime, give a drink of water. Very often it is found that the mother considers her supply of milk insufficient for the wants of the child, and gives, as additional nourishment, arrow-root, corn starch, etc. I believe such food to be extremely improper for a child under six months of age, or at least before it has acquired teeth. The infant has been provided by Nature with the power to digest milk only, prior to this period. Before the eruption of the teeth has begun, there is little or no secretion from the salivary glands, the fluid of which is necessary for the physiological digestion of amylaceous food; and, although the secretion of the mucous membranes in the *adult* may act upon starch in the same manner as the salivary fluid, yet, when the starch is in larger proportion and already within the delicate stomach of the infant, it is very unlikely to be properly digested by that organ, and consequently perverts the action of the intestines in their effort to assimilate it. Whatever may be the *modus operandi* of its perturbating influence, such high authorities as West, Chambers, and Meigs and Pepper all agree as to its impropriety as an article of food for young infants. Hence, I always strenuously interdict such injurious substitution for the natural aliment. It is almost beyond belief to what an extent this method of substitution is prevalent. Even with infants entirely deprived of the breast, and with those of a few weeks old, it is a common practice amongst the lower orders to stuff the little innocent with corn starch *ad nauseam!* I order such practice to be immediately discontinued, and direct the use of cow's milk, diluted, or, what I have found perfectly satisfactory, if not far preferable, condensed milk. The latter is diluted with tepid water, in the proportion of a tablespoonful of the condensed milk to a half pint of water, and a pinch of table salt added. I have seen a child deprived of the mother's breast, raised in this manner on condensed milk, without ever having an attack of indigestion during the whole period of artificial nursing. The child is now over two years old, and is strong and hearty. The great advantage of this plan of artificial nursing is, that the child's food, by a little attention, is always of uniform strength, is easily prepared, and can be used always fresh, and is even beyond the influence of nervous and other physical disturbances on the part of the mother, which is well known may affect the digestion of the child at the breast. There is no doubt but that such improper food as before mentioned, administered to infants, is a fruitful cause of a large number of diarrhœas which may eventually assume all the symptoms and results of a fatal marasmus. I omit any mention of indigestion and its treatment as occurring in children after dentition, as that does not relate to our subject.

In cases of simple diarrhœa occurring in teething children, where there is no fever present, and absence of pain on pressure over the abdomen, where the stools are more frequent, thinner and copious than usual, when the cause is presumed to be nothing more than the highly irritable state of the nervous system, the effect of the transitionary process of teething, and reflecting its action on the alimentary canal, if the gums should be found swollen, red and tender, they would be lanced; but since my term of service at the dispensary, I have never found occasion to lance a gum. I generally, in such cases, administer a sedative, such as the bromide of potassium, in doses of two to four grs. every three hours, and insist upon careful attention to the diet of the child. If an astringent is found necessary, I give mist. cretæ, fʒi; tr. catechu and tr. opii camph., aa. gtts. iv.; every three hours. When the patient has acquired a mixed dietary and presents the same symptoms of simple diarrhœa as before mentioned, the case being due to eating of improper food, if seen shortly after the commencement of the attack, a mixture of ol. ricini and syr. rhei arom., aa f℥ ss.; sodæ bicarb., grs. i.–ii., is ordered to be taken every half hour until the bowels have been freely evacuated, and afterwards to be taken twice or three times daily. This mixture is similar to the Chaussier mixture, with the exception of the soda, which is added as an antacid. Stillé, in his work on Materia Medica, remarks of the ol. ricini, that it is peculiarly adapted to the diarrhœas of children, from causes as at present under consideration; because, while it impresses the general system very slightly, it has a sanitive influence upon the bowels themselves.

In cases of simple diarrhœa occurring in children who have completed the first dentition, where there appears to be a lack of tone in the digestive organs, and where the stools present the condition of lientery, a tonic of quiniæ sulph. and tr. ferri chlorid. is given, together with eight to ten grs. of pepsin, taken with the food at mealtime. Pepsin is also given in those cases of simple diarrhœa, in growing children, where the stools are large, watery, frothy and of fetid odor.

Where simple diarrhœa is met with in strumous children, I administer the following: ol. morrhuæ, ℥ iij.; syr. prun. virg., liq. calcis aa, ℥. i. M. S. One to two teaspoonfuls after each meal. The lime water acts as an emulsifier, and the wild cherry renders the oil more tolerant to the stomach, and at the same time serves to disguise its taste. I have always found the oil to be easily digested after continuing its use for several days, and the looseness of the bowels to gradually disappear without further treatment. When change of temperature, commonly termed cold, is the cause of the diarrhœa, by some writers styled *intestinal catarrh*, a mixture of tr. opii camph., gtts. iv.; ext. ipecac. fld., gtts. ¼, given in a teaspoonful of equal parts of syrup and water, is prescribed for infants, and larger doses for older children. The castor oil mixture answers fully as well in such cases, and is more frequently given than the first-mentioned combination.

I now come to the consideration and treatment of that variety of the Intestinal Disorders of Children that is by far the most frequently met with by the profession, in any portion of this country.

It is the *summer complaint*, by some confounded with cholera infantum, but which I, followlng the able distinction made by our American authorities, Meigs and Pepper, will term entero-colitis, or inflammatory diarrhœa of children.

When this form of diarrhœa presents itself to my notice, appearing in a child undergoing dentition, where the evacuations are frequent and present the familiar green or chopped-spinach appearance, (which, according to such high authority as Chambers, is due to nothing more nor less than blood which has undergone transformation), and also containing mucus and undigested curd—all more or less certain indications of inflammatory destruction; and when, moreover, during the first days of the complaint, it is attended with marked fever and tenderness upon pressure over the abdomen, and more especially in the region of the iliac fossæ, I at once place the little sufferer upon a genuine antiphlogistic treatment, consisting of liq. ammon. acet., or liq. potass. cit., gtts. xx.; tr. opii camph., gtts. iv.–x.; ext. ipecac fld., gtts. $\frac{1}{4}$–$\frac{1}{2}$, given in a teaspoonful of anisette water. I order the diet to be carefully regulated, the breast to be given not oftener than every three hours, and if there be much vomiting, teaspoonful doses of toast-water, containing ice, to be given. In cases where vomiting appears as the principal symptom, I am in the habit of giving the following: hydrarg. chlor. mit., gr. i.; sacch. albæ, gr. xv. M. et in chart No. xvi. div. One to be given every two hours. When the disease has progressed for several days, until the febrile symptoms have subsided, or where such changes appear in the evacuations, as before remarked, following a previous simple diarrhœa, I employ the following powder: pulv. rhei, grs. vj.; pulv. ipecac. co., grs. x.; sodæ bicarb., grs. xij. M. et in chart No. xij. div. One to be given every three hours to a child under one year of age. I also sometimes use the following, for the same age: Vin. ipecac, gtts. ij.; tr. calombæ, gtts. xx.; mist. saline f℥ij. M. To be given every three hours. The mist. saline is made by adding lemon juice in sufficient quantity to neutralize twenty grains of carbonate potassa dissolved in f℥i. water (The composition of the prescription is due to Pavy.) In addition to drugs and attention to diet, I generally recommend a hot bath to be given twice daily, and the baby to be wrapped in a blanket, after being dryed, so as to invite free perspiration. Plenty of fresh air is advised, which is especially necessary in a large city like New York.

When this variety of diarrhœa presents itself in children over a year old, and in those under that age also, where there are streaks of unaltered blood in the stools, I use the following: Bismuth subnit., ʒi; pulv. ipecac. co., gr. xx.; pulv. zinzib., grs. iij. M. et in chart No. xij. dir. One to be given every three or four hours. The bismuth also serves to quiet the stomach where there is much irritability present. When the stools contain undigested matter, I give, in addition to the above, eight to ten grains of pepsin three times daily.

Where the disease has lasted for several months, and has assumed all the features of a chronic diarrhœa, whether the patient has completed dentition or not, I give the cod liver oil mixture before mentioned, in the proportion of fʒ iii. ss. to fʒ ss. of the syr. ferri iodid., a teaspoonful of which is so be given three times daily. It acts in the same beautiful and pleasing manner as in the simple diarrhœa of strumous children. Indeed, there is an analogy between the two affections, as in both, the mesenteric glands are enlarged, and these, no doubt, are important factors in the chronicity of the disease. I have sometimes advised the raw-beef diet to be used in chronic diarrhœa, but it has been more from wishing to vary my practice than from any want of confidence in the cod liver oil and syrup of the iodide of iron.

Cholera infantum, properly so called, is a disease which is not so frequently met with, unless there exists some epidemic influence. So far, in my dispensary practice, I have not seen over a dozen cases of it in two years. In order to be explicit in my discrimination of the treatment of cholera infantum, I will premise by remarking, that I refer to such cases as are accompanied with profuse watery evacuations, altogether different in appearance from the stools oı entero-colitis, severe vomiting, great thirst, suppression of urine, fóllowed by collapse and convulsions. I scarcely think such cases can be confounded with entero-colitis, by an observant physician, and I make this remark, as the treatment which I have used might not be so applicable in cases where no choleraic element was present.

Looking upon the cholera of infants as due to the same cause as that of the adult, only in a modified degree, namely, the poisoning of the blood by the presence of fungi which have been developed through the influence of heat, filth and moisture, I have always prescribed the carbolic acid, regarding this agent as especially destructive to all fungi, whether in or out of the blood. The marked success that I have had in the treatment of cholera infantum, justifies the result of my theory. Though I have not had any great number of cases of this disease to treat, yet all of my cases have responded promptly to the treatment, except one that died on the same day that I saw it, and could hardly be called a fair case for treatment. In one that I remember, which was one of my first cases, after using the carbolic acid for two days, the case was considered cured, and discharged, all evacuations having ceased for the time. But after two more days the case was returned, with the same symptoms as at first, and the same remedy being used, it again responded as promptly to the treatment as before. I afterwards used an astringent preparation for a week or so until the case was beyond any further danger of a relapse. I thus learnt that, although the carbolic acid acts speedily, it is apt to deceive the physician, and he must be on his guard against a relapse; so that, since my first case of cholera infantum, I always administer an astringent, even after all choleraic symptoms have disappeared, for a week or ten days. The formula which I use is as follows: Acid. carbolic. (chryst.) *Calvert's*, grs. ij.; glycerine, fʒ i.; aquæ, fʒ vij. M. Teaspoonful every two hours. It checks the vomiting as speedily as it does the watery evacuations. I always allow plenty of ice-water as a drink, and advise the use of lime-water, and milk, and weak beef tea as food. In regard to the treatment of the stage of collapse, I have very little to say, from experience. I would suggest the carbolic acid treatment in connection with small doses of spts. ammon. arom., or liq. ammon. acet., or small doses of old brandy, together with warm applications to the feet and hands. Some of our physicians recommend warm applications to the head also, even during a convulsion, which appears to me as rational treatment. The warm bath, in the first stage, is advised by some authors on the subject.

I have only a few remarks to make concerning the treatment of dysentery in children. Nearly all of the cases that I have seen were secondary, following attacks of measles. The stools consisted of mucus and blood, and resembled the washings of finely minced meat. The treatment I used was bismuth and Dover's powder, in the same doses as before given under the subject of entero-colitis. As there are poor opportunities to administer enemata in dispensary practice, from the uncertainty of trusting the performance into ignorant hands, I have not used them. If an opportunity should offer where they could be correctly given, I should advise the use of either the nitrate of silver in small doses, or the sulphate of zinc with tr. opii, in proper quantities.

I have thus, gentlemen, endeavored to give you a concise *resumé* of my practice in this class of children's diseases. As regards the succese of my treatment as I have given it to you, I have only the following method of judging: All patients coming to the dispensary are registered, and the mothers are given cards with a number thereon corresponding to the name on the register. The mothers are requested to return, to report the result of the treatment from time to time, and many do so. There are others, however, who do not report, unless in case of the death of the patient, when they are sure to come for a certificate of death, that has to be filed in the register's office of the health department, in order to procure a burial permit. So, we are always certain to know if our patient dies; and if they do not die and do not return to report, we take it for granted that the treatment has been successful. I am sure that I have not lost a dozen cases from intestinal diseases out of many hundreds that I have treated in the last two years.

52 *Sixth Avenue, New York, Feb.* 15, 1873.

---

USE OF ARSENIC IN THE TREATMENT OF CONSTIPATION.—M. Isnard recommends arsenic in the treatment of constipation, especially in the case of females. Although any preparation of arsenic seems to answer, M. Isnard prefers, ordinarily, arsenious acid. Three grains being dissolved in two and a half pints of water, a coffee-spoonful will represent one milligramme of the acid. A medium dose is six to ten coffee-spoonsful daily, in three doses, preferably taken at meal-time, in water or wine, and increased gradually in quantity.—*La France Médicale.*

